# 10-Year natural course of early hip osteoarthritis in middle-aged persons with hip pain: a CHECK study

**DOI:** 10.1136/annrheumdis-2020-218625

**Published:** 2021-01-15

**Authors:** Annemaria C. van Berkel, Dieuwke Schiphof, Jan H. Waarsing, Jos Runhaar, John M. van Ochten, Patrick J.E. Bindels, Sita M.A. Bierma-Zeinstra

**Affiliations:** 1 Department of General Practice, Erasmus MC, University Medical Center, Rotterdam, The Netherlands; 2 Department of Orthopaedics, Erasmus MC, University Medical Center, Rotterdam, The Netherlands

**Keywords:** osteoarthritis, epidemiology, patient reported outcome measures

## Abstract

**Objective:**

To explore the natural course of hip osteoarthritis (OA) in a population of first-time presenters with hip complaints.

**Methods:**

Data were collected at baseline and after 2, 5, 8 and 10 years on participants from the Cohort Hip and Cohort Knee study with early symptomatic hip OA. Descriptive statistics were used to analyse the natural course of the hip complaints with respect to clinical signs and symptoms, physical functioning and radiographic osteoarthritis (ROA) features.

**Results:**

In total, 588 participants were included with hip complaints and 86% completed the 10-year follow-up. The 10-year follow-up showed that 12% (69 participants) underwent hip replacement (HR), an increase of ROA of the hip (Kellgren and Lawrence score≥2) from 19% to 49%, and an increase in clinical hip OA according to the American College of Rheumatology criteria from 27% to 43%. All Western Ontario and McMaster Osteoarthritis Index subscales and physical activity remained on average constant during the 10-year follow-up for those who did not undergo an HR. The use of pain medication increased from 43% at baseline to 50% after 10 years.

**Conclusion:**

One out of nine participants with early hip problems received an HR during the 10-year follow-up. Prevalence of clinical hip OA and hip ROA increased steadily during the 10-year follow-up. Overall, we observed more hip OA, but fewer or stable complaints with respect to clinical signs and symptoms, and physical functioning. So it could be cautiously concluded that after 10 years, first-time presenters with hip complaints either received an HR or their symptoms remained stable.

**Key messages**

**What is already known about this subject?**

- The natural course of hip complaints that may be indicating hip osteoarthritis (OA) over time is still poorly characterised.

**What does this study add?**

- This study provides long-term (10-year) follow-up data about the clinical signs and symptoms of hip OA in people with hip pain.
- One out of nine participants with early hip problems underwent a hip replacement during the follow-up.
- Prevalence of clinical hip OA and radiographic hip OA increased steadily during the 10-year follow-up, but complaints remained stable.

**How might this impact on clinical practice or future developments?**

- This study provides more information on long-term outcomes to determine the course of progression of hip OA.

## Introduction

Osteoarthritis (OA) of the hip is a common problem in Western society and a common diagnosis in primary care.^1^ Hip OA affects 7%–25% of people older than 55 years.^2^ The number of affected hips will increase with further ageing of the population.^2^ Pain in the hip and hip stiffness are the most common symptoms of hip OA.^3^ Consequently, patients are restricted in their activities, which has an impact on the health-related quality of life. For a disease so common and with an enormous impact on the affected patients, remarkably little is known about the natural course of early signs of hip OA. Most previous studies investigated the natural course of hip complaints combined with knee complaints.^4 5^ Knee OA is more common than hip OA and has been much more often studied.^6^ As a result, the natural course of hip complaints that may be indicating hip OA over time is still poorly characterised.

For many patients, the primary care physician (PCP) is the first physician they consult in their hip OA process. Therefore, it is important to investigate the natural course of hip complaints so that the PCP can start the most relevant non-surgical management, and inform what to expect. The aim of the present study was to describe the natural course of hip complaints with respect to clinical signs and symptoms, physical functioning and radiographic osteoarthritis (ROA) features during the 10-year follow-up of middle-aged first presenters with hip complaints.^7^

## Patients and methods

### General design

The data for this study were acquired from the Cohort Hip and Cohort Knee (CHECK) study; details on this cohort have been published elsewhere.^8^ In short, the CHECK study is a prospective, 10-year follow-up cohort in the Netherlands of 1002 first presenters with hip and/or knee pain. Individuals entered the cohort between October 2002 and September 2005. Inclusion criteria for the CHECK study were (1) stiffness and/or pain in the hip and/or knee, (2) age of 45–65 years, and (3) participants who had not yet consulted their PCP for these symptoms or (4) the participants’ first consultation was within 6 months of entry. Exclusion criteria were (1) other pathological conditions that could explain the existing complaints (eg, other rheumatic disease, previous hip/knee joint replacement, congenital dysplasia, osteochondritis dissecans, intra-articular fractures, septic arthritis, Perthes' disease, ligament or meniscus damage, plica syndrome and Baker’s cyst); (2) comorbidity that would not allow physical evaluation during the follow-up; (3) malignancy in the past 5 years; and (4) inability to understand the Dutch language.

### Study population

We included participants reporting hip pain at baseline. All participants were divided into subgroups twice; the first subgroups were a subgroup who reported hip pain (yes/no) only at baseline (H group) and a subgroup who reported hip and knee pain (yes/no) at baseline (H&K group). Subsequently, all participants (regardless of whether they reported hip and knee pain at baseline) were divided into a second subgroup based on whether they underwent a hip replacement (HR) during follow-up (HR group) or did not receive an HR (no-HR group).

### Outcome variables

Information about pain and other symptoms, physical functioning, education level, height and weight (to calculate body Bass Index (BMI)), comorbidity, quality of life and psychosocial factors was collected at five different points (at baseline, after 2 years (T2), T5, T8 and T10). The information was collected by means of self-reported questionnaires and a physical examination. The Western Ontario and McMaster Osteoarthritis Index (WOMAC) was used to measure pain, stiffness and physical functioning (higher score indicating worse health). Pain intensity was assessed using the Numerical Rating Scale (NRS) for pain intensity (range 0–10, higher scores indicating more pain). The participants were asked to score the pain intensity they experienced in their most painful joint over the past week and to score the present pain intensity in the same joint. At T5, T8 and T10, the participants were asked to report pain intensity related to the left and right hips for the past week. Of these measurements, we used the highest scores as the pain intensity outcome. In the physical examination, hip pain during flexion of the joint (yes/no) and pain during internal rotation of the hip (yes/no) were recorded. In addition, patients were asked whether they had morning stiffness in the hip (yes/no) and if they had hip and knee pain (yes/no).

At baseline, T2, T5, T8 and T10, standardised radiographs were collected of the anteroposterior view, pelvis view or unilateral faux profile view of both hips and of the tibiofemoral joints (both knees). The radiographs were centrally scored for OA features^9^ according to the Kellgren and Lawrence (K/L) criteria^10^ and for OA features according to criteria described by Altman and Gold.^11^ In the hip, all radiograph features showed good interobserver reliability.^9^ Radiographic hip or knee OA was defined as K/L grade≥2.^12^ Information on HR and/or knee replacements was obtained from radiographs. Clinical hip and knee OA were determined according to the criteria of the American College of Rheumatology (ACR), which for the hip are hip pain and all of the following criteria under 1 or 2: (1) hip internal rotation of ≥15°, pain present on internal rotation of the hip, morning stiffness (≤60 min) and aged >50 years; (2) hip internal rotation of <15° and hip flexion of ≤115°.^13^ The ACR criteria for clinical knee OA are knee pain and ≥3 of the following symptoms: (1) aged >50 years, (2) morning stiffness (<30 min), (3) crepitus on active motion, (4) bony tenderness, (5) bony enlargement and (6) no palpable warmth of synovium.^14^ If a participant fulfilled these clinical ACR criteria at least once during follow-up, they were classed as a clinical hip/knee OA participant.

### Statistical analysis

Descriptive statistics were used to analyse the baseline characteristics and the course of the variables. The last observation carried forward (LOCF) from the last visit prior to HR was used for a subanalysis in the HR group to explore the course of symptoms if, as a thought experiment, HR had not been available (HR group with LOCF). Statistical analyses were performed using SPSS V.24.0 for Windows.

## Results

### General characteristics

In total, 588 of the 1002 participants reported hip pain at baseline. Of these 588 included participants, 170 participants (29%) reported only hip pain, 418 participants (71%) reported both hip and knee pain and 81 participants were lost to follow-up (online supplemental figure 1). Table 1 summarises the characteristics of the study population at baseline. At baseline, the mean age was 55.8 (SD=5.2) years; the mean BMI was 26.1 (SD=4.1) kg/m^2^; and 81% was female. Most prominently, it shows that 19% of the participants had ROA of the hip, and 27% of the participants met the clinical ACR criteria for hip OA. More participants of the H group met the clinical hip OA criteria (30%) and showed ROA (23%) compared with participants of the H&K group at baseline (26% and 17%, respectively). During follow-up, 249 participants (43%) fulfilled the criteria at ≥1 assessment. At baseline, 48 out of 160 participants (30%) with clinical hip OA also showed ROA in at least one hip.

10.1136/annrheumdis-2020-218625.supp1 Supplementary data

**Table 1:** Baseline characteristics (mean (SD) or number (%)) of the study population and the subgroups

Baseline characteristics/factors	Total study population	H group	H&K group	HR group	No-HR group
Number of participants	588	170	418	69	518
Age (years), mean (SD)	55.8 (±5.3)	55.7 (±5.6)	55.8 (±5.2)	57.4 (±4.8)	55.6 (±5.3)
Female, n (%)	475 (81)	129 (76)	346 (83)	48(70)	426 (82)
Caucasian, n (%)	578 (99)	169 (99)	409 (98)†	68 (100)	509 (98)
Body Mass Index (kg/m^2^), mean (SD)	26.1 (±4.1)†	25.5 (±3.5)†	26.4 (±4.2)†	25.8 (±3.8)†	26.2 (±4.1)†
Education level, n (%) Primary Secondary Higher	†107 (19) 267 (47) 199 (35)	†25 (15) 776 (46) 65 (39)	†82 (20) 191 (47) 134 (33)	†16 (24) 33 (49) 18 (27)	†91 (18) 233 (46) 181 (36)
Never smoked, n (%)	175 (30)†	56 (34)†	119 (29)†	29 (43)†	146 (29)†
No use of alcohol, n (%)	125 (22)†	30 (18)†	95 (23)†	17 (25)†	108 (22)†
Use of any pain medication, n (%)	250 (43)†	63 (38)†	187 (46)†	29 (43)†	221 (44)†
Three or more comorbidities, n (%)	152 (26)†	33 (20)†	119 (29)†	5 (7)†	147 (29)†
Baseline NRS (0–10) past week, mean (SD)	3.7 (±2.1)†	3.4 (±2.2)†	3.8 (±2.1)†	4.1 (±2.4)†	3.6 (±2.1)†
Baseline NRS (0–10) present pain, mean (SD)	3.2 (±2.1)†	2.8 (±2.0)†	3.4 (±2.1)†	3.8 (±2.4)†	3.2 (±2.0)†
Morning stiffness in the hip <60 min, n (%)	326 (55)	101 (59)	225 (54)	43 (62)	282 (54)
Knee pain, n (%)	418 (71)	0 (0)	418 (100)	31 (45)	386 (75)
Physically active (>30 min) for three or more times a week, n (%)	316 (55)†	103 (62)†	213 (53)†	34 (52)†	282 (56)†
WOMAC, mean (SD) Pain (0–20) Stiffness (0–8) Physical function (0–68) Total sum score (0–100)	5.4 (±3.4)† 2.8 (±1.7)† 17.2 (±12.0)† 26.4 (±16.8)†	4.8 (±3.2)† 2.5 (±1.7)† 14.7 (±11.1)† 22.6 (±15.4)†	5.7 (±3.5)† 2.9 (±1.7)† 18.3 (±12.2)† 28.0 (±17.1)†	5.7 (±3.9)† 2.9 (±1.6)† 20.1 (±12.5)† 29.9 (±17.9)†	5.4 (±3.4)† 2.8 (±1.7)† 16.9 (±11.9)† 26.0 (±16.6)†
Radiographic severity K/L grade ≥2 either hip, n (%)	110 (19)†	38 (23) †	72 (17)†	37 (54)	73 (14)†
Radiographic severity K/L grade ≥2 either knee, n (%)	76 (13)†	14 (8)†	62 (15)†	12 (17)	64 (13)†
Clinical hip OA,* either hip, n (%)	160 (27)†	51 (30)†	109 (26)†	34 (49)	125 (24)†
Clinical knee OA,* either knee, n (%)	206 (35)†	0	206 (50)†	12 (17)	194 (38)†
Physical examination, n (%) Painful internal rotation, either hip, n (%) Painful flexion, either hip, n (%)	322 (55)† 315 (54)†	101 (60)† 94 (56)†	221 (53)† 221 (54)†	50 (73) 48 (71)†	271 (53)† 267 (52)†

Values are mean value±SD or percentages (%).

Participants can be part of two subgroups, for example, H group and HR group.

*According to the clinical criteria of the American College of Rheumatology.

†≤4.3% missing.

H, subgroup who reported hip pain only at baseline; H&K, subgroup who reported hip and knee pain at baseline; HR, group who underwent a hip replacement during follow-up; K/L, Kellgren and Lawrence; NRS, Numerical Rating Scale; WOMAC, Western Ontario and McMaster Osteoarthritis Index.

### Clinical and radiographic hip OA during follow-up

**Figure 1:**
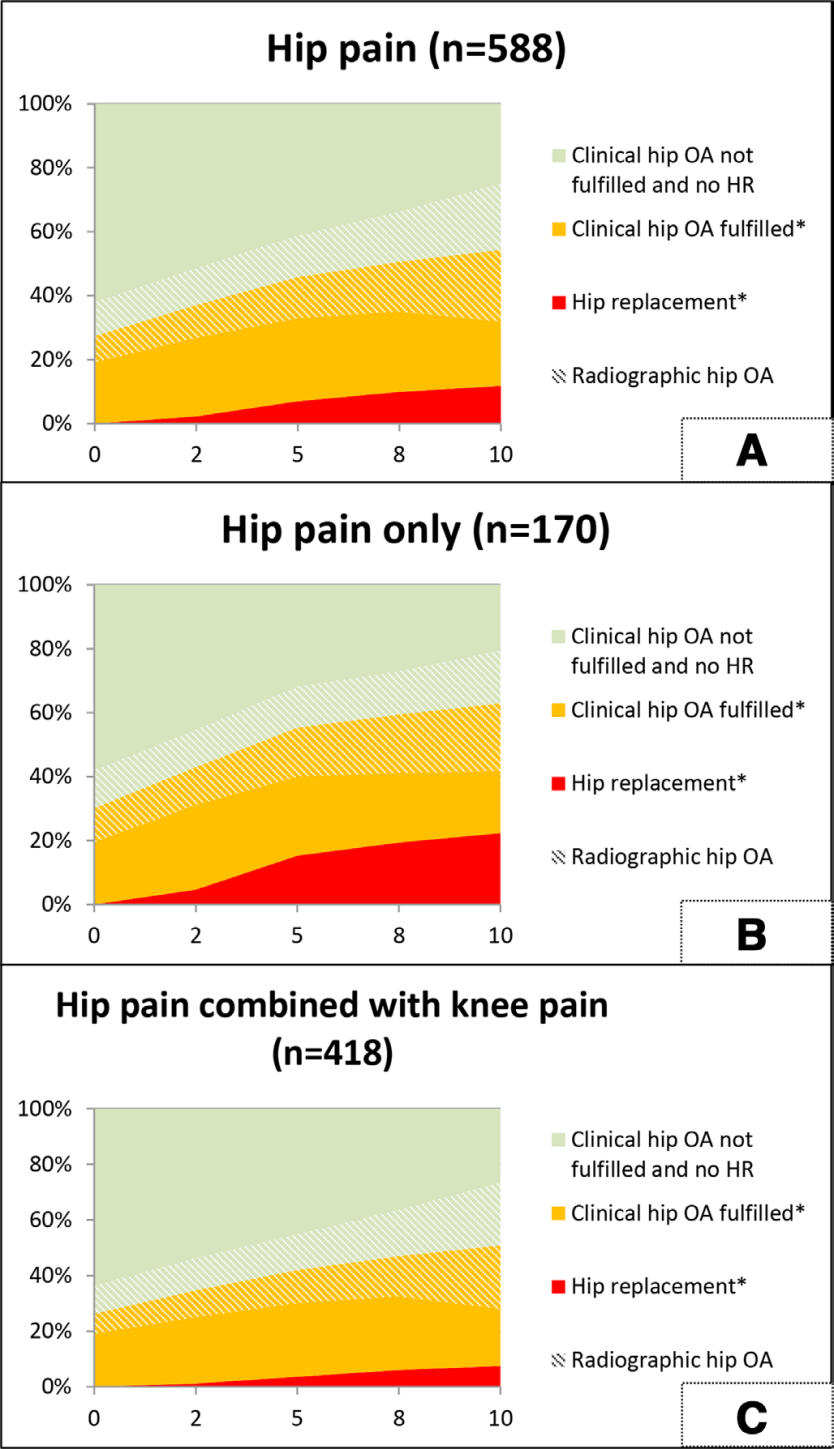
Overview of percentages of participants (reported hip pain at baseline (A), reported only hip pain at baseline (B) and reported both hip and knee pain at baseline (C)) with hip replacement, clinical hip OA according to the ACR criteria and radiographic hip OA according to Kellgren and Lawrence score ≥2 over 10 years of follow-up. *cumulative. A: at t0 4 missing participants, at T2-T10 3 missing participants. B: at t0 1 missing participant. C: at t0 -T10 3 missing participants. OA, osteoarthritis.

After 10 year, 131 out of 249 participants (53%) with clinical hip OA had ROA in at least one hip. Of the participants without clinical hip OA at baseline, 62 out of 424 (15%) showed ROA in at least one hip at baseline and after 10 year 122 out of 267 (46%) did so. Most HRs (58 out of a total of 69) took place between T2 and T8. Figure 1A shows the course of these outcomes, taking into account that a participant can only belong to one outcome group at each time point. During follow-up, clinical hip OA and ROA increased and more people received HRs (figure 1A).

Compared with participants of the H&K group, participants of the H group were as likely to meet the clinical hip OA criteria after follow-up (41% vs 43%) and were as likely to show ROA (48% vs 49%). After 10 years, participants of the H group were more likely to undergo an HR compared with participants of the H&K group (22% vs 7%). Figure 1B, C show the course of these outcomes, taking into account that a participant can only belong to one outcome group at each time point.

### Clinical signs, symptoms and physical functioning during the 10-year follow-up

Table 2 and online supplemental table 1 summarises the findings from 10 years follow-up. After 10 years, only 51% still reported hip pain and fewer participants reported morning stiffness in the hip (55% at baseline vs 45% after 10 years) than at baseline. In comparison with baseline, lower hip pain intensity over the past week was observed (3.7 (SD=2.1) vs 2.9 (SD=2.6)). Slight differences between baseline and the follow-up were observed in all WOMAC subscales. The use of any pain medication increased during follow-up (43% at baseline vs 50% after 10 years). The number of individuals who were physically active (>30 min≥3 times/week) stayed stable over time (55% at baseline vs 56% after 10 years). During follow-up, the number of participants with clinical hip OA increased, as well as the participants with clinical knee OA (table 2). We observed a decrease in painful movements of the hip: painful internal rotation, external rotation and flexion of the hip are reported more frequently at baseline compared with the follow-up (table 2). Compared with participants of the H group, participants of the H&K group reported slightly higher scores for pain (intensity), stiffness and physical function during follow-up (online supplemental table 1).

**Table 2:** Course of pain, physical functioning and radiographic OA features during follow-up, for total study group (n=588)

	Baseline	T2	T5	T8	T10
WOMAC, mean (SD) Pain (0–20) Stiffness (0–8) Physical function (0–68)	5.4 (±3.4) 2.8 (±1.7) 17.2 (±12.0)	5.2 (±3.5) 2.6 (±1.6) 16.4 (±12.0)	5.1 (±4.0) 2.8 (±1.8) 17.6 (±12.0)	4.5 (±3.6) 2.4 (±1.8) 16.3 (±13.0)	4.7 (±3.8) 2.6 (±1.9) 16.2 (±13.1)
NRS past week, mean (SD)	3.7 (±2.1)	3.7 (±2.3)	3.2 (±2.6)	2.7 (±2.5)	2.9 (±2.6)
Use any pain medication, n (%)	250 (43)	263 (48)	251 (47)	250 (49)	249 (50)
Hip pain, n (%)	588 (100)	374 (68)	301 (57)	267 (54)	247 (51)
Knee pain, n (%)	418 (71)	361 (65)	327 (62)	297 (58)	264 (53)
Morning stiffness (hip) <60 min, n (%)	326 (55)	287 (52)	272 (51)	239 (47)	228 (45)
Physically active (>30 min) for ≥3 times a week, n (%)	316 (55)	319 (60)	292 (56)	296 (58)	276 (56)
Cumulative sum of HR, n (%)	0 (0)	13 (2)	41 (7)	58 (10)	69 (12)
Cumulative sum of KR, n (%)	0 (0)	0 (0)	4 (1)	9 (2)	10 (2)
K/L grade ≥2 either hip, n (%)	110 (19)	128 (22)	151 (28)	183 (35)	253 (49)
Clinical knee OA* either knee, n (%)	206 (35)	278 (47)	323 (55)	349 (59)	366 (62)
Painful internal rotation either hip, n (%)	322 (55)	197 (36)	190 (38)	166 (36)	179 (39)
Painful external rotation either hip, n (%)	160 (35)	86 (17)	115 (20)	86 (15)	89 (20)
Painful flexion either hip, n (%)	315 (54)	227 (42)	192 (39)	149 (32)	159 (35)

Values are mean value±SD, or number (percentages, %).

*According to the clinical criteria of the ACR; once those clinical ACR criteria are satisfied, the case will be seen as clinical hip or knee OA.

ACR, American College of Rheumatology; HR, hip replacement; K/L, Kellgren and Lawrence; KR, knee replacement; NRS, Numerical Rating Scale; OA, osteoarthritis; WOMAC, Western Ontario and McMaster Osteoarthritis index.

### Clinical signs, symptoms, physical functioning and HRs

**Figure 2:**
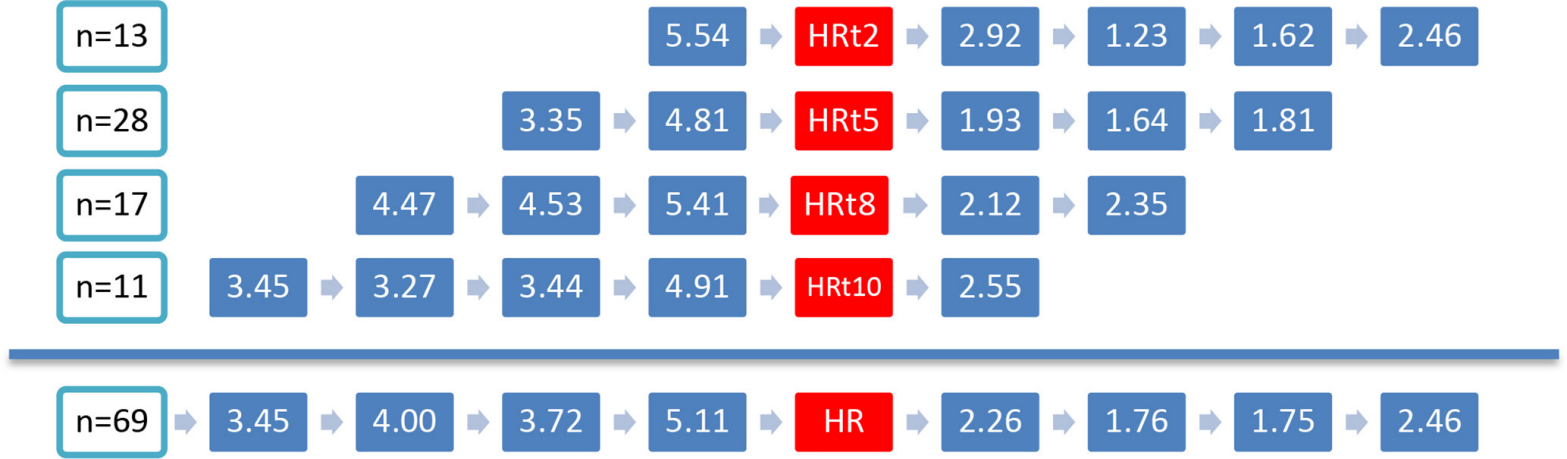
Overview of self-reported NRS pain score in past week for participants who received an HR, represented per time point when they received an HR. Left of the red box (= when HR is seen on radiograph) pain scores before HR with intervals of 2 or 3 years, and right of the red box the pain scores with HR. The last row represents the weighted average pain scores for all participants.

**Figure 3:**
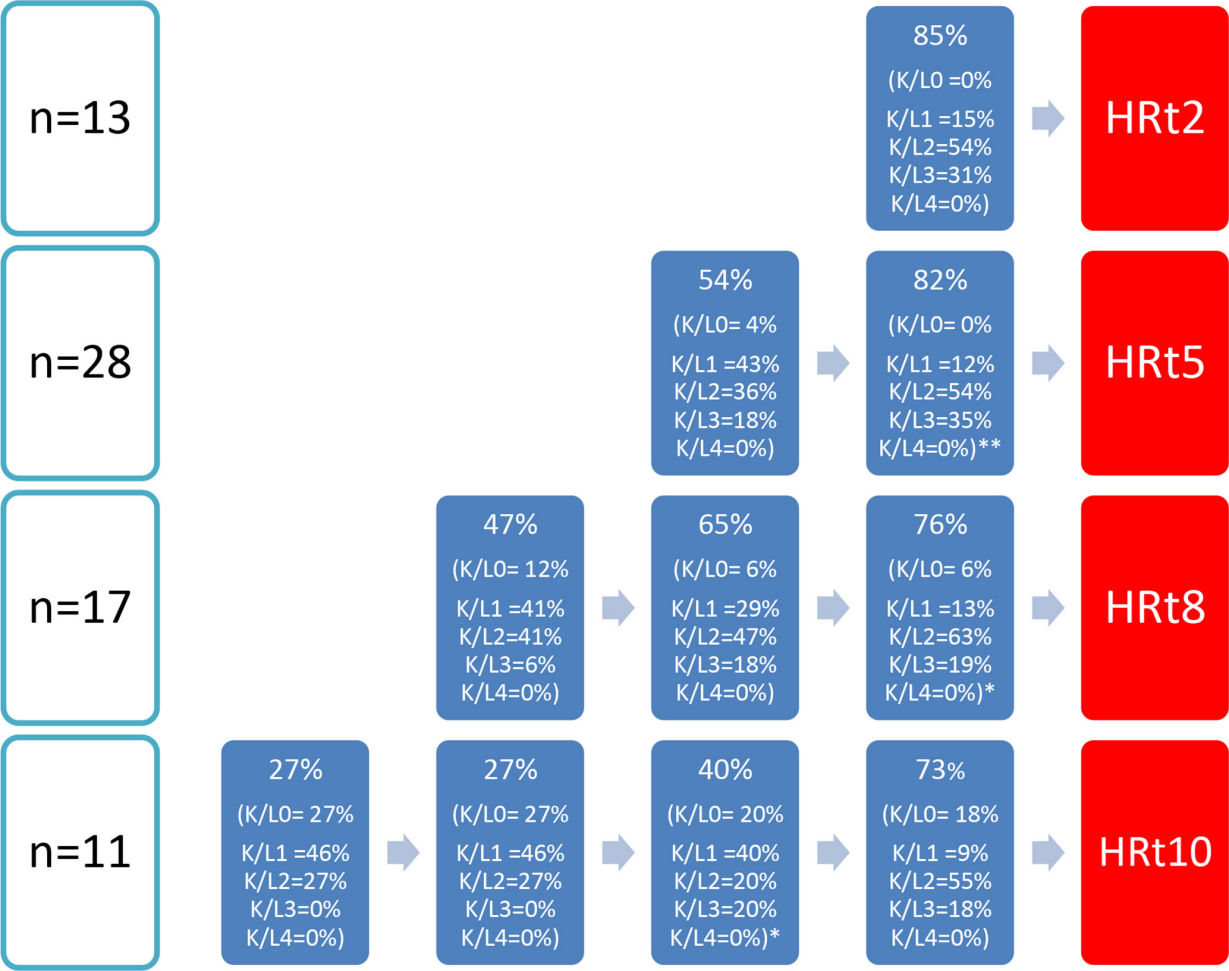
Overview of percentages of participants with radiographic hip OA according to Kellgren and Lawrence score ≥2 and noted in brackets the highest Kellgren and Lawrence score of any hip in time intervals of 2 or 3 years beforeHR. *=1 missing, **=2 missing.

**Figure 4:**
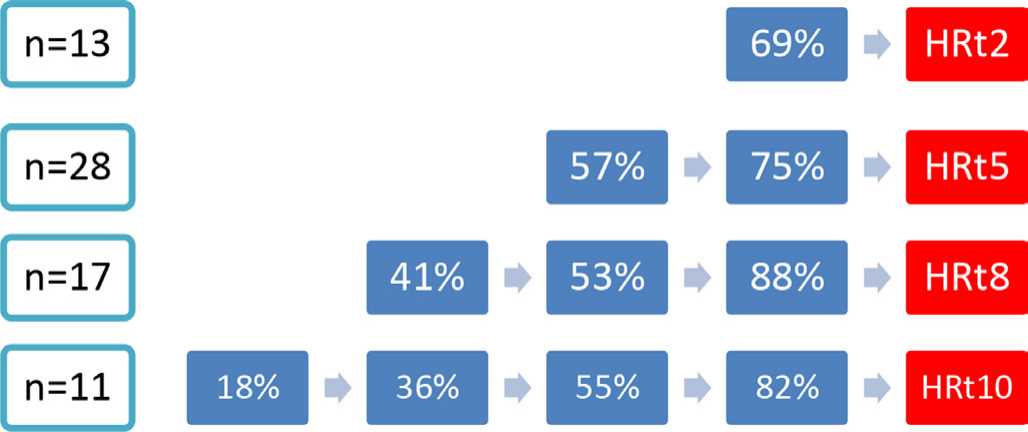
Overview of percentages of participants with clinical hip OA in time intervals of 2 or 3 years before HR.

Online supplemental table 2 summarises the findings for the 10-year follow-up of the HR group and no-HR group. In addition (online supplemental table 2) shows the results for HR group with LOCF. Participants of the HR group have higher prevalence of ROA and were more likely to have met the clinical hip criteria at baseline. After 10 years, participants of the HR group reported lower pain intensity (both on the NRS, −1.9 points after 10 years) and on the WOMAC subscale (−2.6 points after 10 years, in analysis without LOCF) and lower scores for physical function (−8.1 points on WOMAC physical function after 10 years, in analysis without LOCF) compared with baseline. Use of any pain medication after 10 years seemed to decrease in persons of the HR group and increased in the no-HR group. Regarding the physical examination results, we observed an increase in painful movements of the hip in participants of the HR group: painful internal rotation, external rotation and flexion of the hip are reported more frequently after 10 years compared with the baseline (online supplemental table S2). However, at T10, only a small number of participants underwent a physical examination in the HR group. Figure 2 shows the course of (hip) pain intensity over the past week, figure 3 the course of ROA and figure 4 the course of clinical hip OA in the years preceding the HR (for the HR group). We observed higher pain intensity for the past week before HR compared with the pain intensity during the follow-up with an HR (figure 2). The percentage with ROA increased in the years before the HR; only a small proportion of participants had severe ROA (19%–35% K/L 3 and 0% K/L 4 obtained from radiographs before HR) (figure 3). Most participants met the clinical hip OA criteria (69%–88%) before undergoing an HR (figure 4).

## Discussion

We observed that 12% of first-time presenters with hip complaints underwent an HR during 10 years follow-up. Furthermore, the prevalence of both clinical hip OA and ROA increased during follow-up. We observed less participants reporting hip pain, on average stable pain intensity, stable WOMAC pain scores and less reported pain during physical examination after 10 years compared with baseline, for those who did not undergo an HR. On the other hand, more participants were using any pain medication during follow-up. In general, the participants of the HR group had relatively higher pain intensity and higher prevalence of ROA and clinical hip OA before receiving the HR.

The WOMAC subscales and physical activity remained stable over time, which is in line with other longitudinal studies.^15–17^ This may be based on regression to the mean, but it is also possible that at a group level, study participants truly do not get worse over time. Another explanation could be the response shift phenomenon,^18^ indicating that as time goes on, individuals learn to cope with their chronic disease. Many participants with mild OA have worse periods with more complaints followed by better periods with fewer complaints, and a response shift could have occurred in the self-reported questionnaires. Previous studies using trajectories showed that, on average, the majority remained stable, but they also showed that some of the subjects improved and stayed improved.^19 20^ A remarkable result is the decrease in the number of patients reporting hip pain (except for the HR group). This might be due to the fact that all participants started with pain at baseline, and therefore, the number of participant with hip pain could only stay stable (still have pain) or decrease at follow-up measurements. Furthermore, decreasing pain levels during physical examination over a period of 10 years is in line with previous studies^21–23^ and is a logical consequence of less hip pain.

We observed an increased use of any pain medication during follow-up. This increment could be an additional explanation for the stable WOMAC scores and an even decreasing trend in pain during physical examination. More experience in when to use pain medication and a positive response during flares might influence the overall pain intensity in patients with hip OA. This increase in the use of pain medication is in line with other literature. A study that investigated pain medication for knee OA also showed an increased use of paracetamol and non-steroidal anti-inflammatory drug over 3 years of follow-up.^24^ Other studies have shown that the use of (over-the-counter) pain medication among a general population has increased (modestly) over the past decades.^25 26^

In line with a previous study,^27^ we showed that patients with hip pain can have ROA without fulfilling the ACR criteria for clinical hip OA and vice versa. Our findings of the number of participants undergoing an HR are similar to those of other studies; a study with a 6-year follow-up reported rates for receiving an HR of 22%.^28^ This result is even higher than our findings, despite the shorter follow-up, but the mean age and the amount of ROA at baseline were higher in that particular study. Besides the increase in clinical hip OA, we also showed an increase in clinical knee OA in patients with hip pain at baseline. This might indicate that we have included some participants who initially had knee problems, as a recent study suggested that hip flexion and internal rotation might be affected by early knee OA.^29^ Thereby, we defined clinical hip (and knee) OA from the moment participants fulfilled the clinical ACR criteria; however, it is known that patients intermittently fulfilled the criteria over longer follow-up.^20^ It should also be mentioned that the ACR criteria are widely used in epidemiological research but are not validated in primary care.^30^

Twelve percent of the our study population underwent an HR during follow-up. It could be argued that an HR was justified for these participants because of the progression of their pain intensity, and the majority had ROA and/or clinical hip OA preceding the HR. Nevertheless, they still did not have very high levels of pain intensity, and relatively only a small proportion of participants with K/L≥2 had severe ROA. So it could still be considered as mild hip OA. The greatest benefit from joint replacement is expected if the procedure is restricted to patients with more severely affected functional status and more severe ROA.^31^ As shown in online supplemental table 2, it seems unlikely that participants of the HR group are suppressing the scores for the total group, because the outcomes for pain and physical function for our total group and the no-HR group are quite similar.

To our knowledge, this is the first study that provides long-term follow-up data about the clinical signs and symptoms of hip OA in people with hip pain. The strengths of the present study are that it is a population-based prospective longitudinal design, with a large sample of persons with early-stage symptomatic hip OA, monitored from the onset of disease management in primary care and a follow-up of 10 years. A limitation of the study is that, although participants were asked where the pain was located, participants were not asked to fill in the WOMAC questionnaire (at each follow-up moment) and NRS (at baseline and T2) for a specific joint. Therefore, we do not know if the NRS score, measured at baseline and at T2 for the participants who reported both hip and knee pain, was really pain due to hip symptoms. To solve this problem as well as we could, we selected participants with only hip pain at baseline as a subgroup, but still misclassification is possible. A second limitation to our study is that we had follow-up assessments every 2 or 3 years, at which we asked the participants about the pain intensity in the past week. So these results are not representative for the entire follow-up time. Future research with more frequent symptom assessment could solve this problem. Finally, pain could be reduced by other treatments. We do not have information on the specific treatments people had.

This study provided more background information about the natural course of hip complaints during 10 years of follow-up in first presenters with hip complaints. In conclusion, we observed that the prevalence of clinical hip OA and ROA increased. After 10 years of follow-up, one out of nine (11.7%) participants had undergone an HR. Overall, we observed more hip OA, but less participants reported hip pain. Complaints with respect to clinical signs and symptoms and physical functioning remained stable. It could be cautiously concluded that after 10 years, first-time presenters with hip complaints either underwent an HR, or their symptoms remained stable or improved slightly. Further research should aim to investigate how the course of pain intensity in individuals changes over time and what factors are associated with the fluctuation of pain intensity.
