# Supplementary material for: 10-Year natural course of early hip osteoarthritis in middle-aged persons with hip pain: a CHECK study
Source: Ann Rheum Dis. 2021 Jan 15;80(4):487–93. doi: 10.1136/annrheumdis-2020-218625 (PMC7958083; doi:10.1136/annrheumdis-2020-218625)
Supplement: Supplementary data [file annrheumdis-2020-218625supp001.pdf]

Ten-year natural course of early hip osteoarthritis in middle-aged persons with hip pain; a CHECK study

Online supplementary material

A.C. van Berkel, MD[1], D. Schiphof, PhD[1], J.H. Waarsing, PhD[2], J. Runhaar, PhD[1], J.M. van Ochten, PhD[1], P.J.E. Bindels PhD, professor[1], S.M.A. Bierma-Zeinstra, PhD, professor[1,2]

1 Department of General Practice, Erasmus Medical Center, Rotterdam, The Netherlands, 2 Department of Orthopaedics, Erasmus MC, University Medical Center, Rotterdam, The Netherlands

Supplementary Figure S1	Flow-chart indicating the available data per time point.
Supplementary Table S1	Course of pain, physical functioning, and radiographic OA features during follow-up, for total study group (n=588) and subdivided for H-group (n=170) and H&K-group (n=418).
Supplementary Table S2	Course of pain, physical functioning, and radiographic OA features during follow-up, for the no HR-group (n=518) and HR-group (n=69) and for HR-group with LOCF.
Supplementary Table S3	Course of pain, physical functioning, and radiographic OA features during follow-up with LOCF.

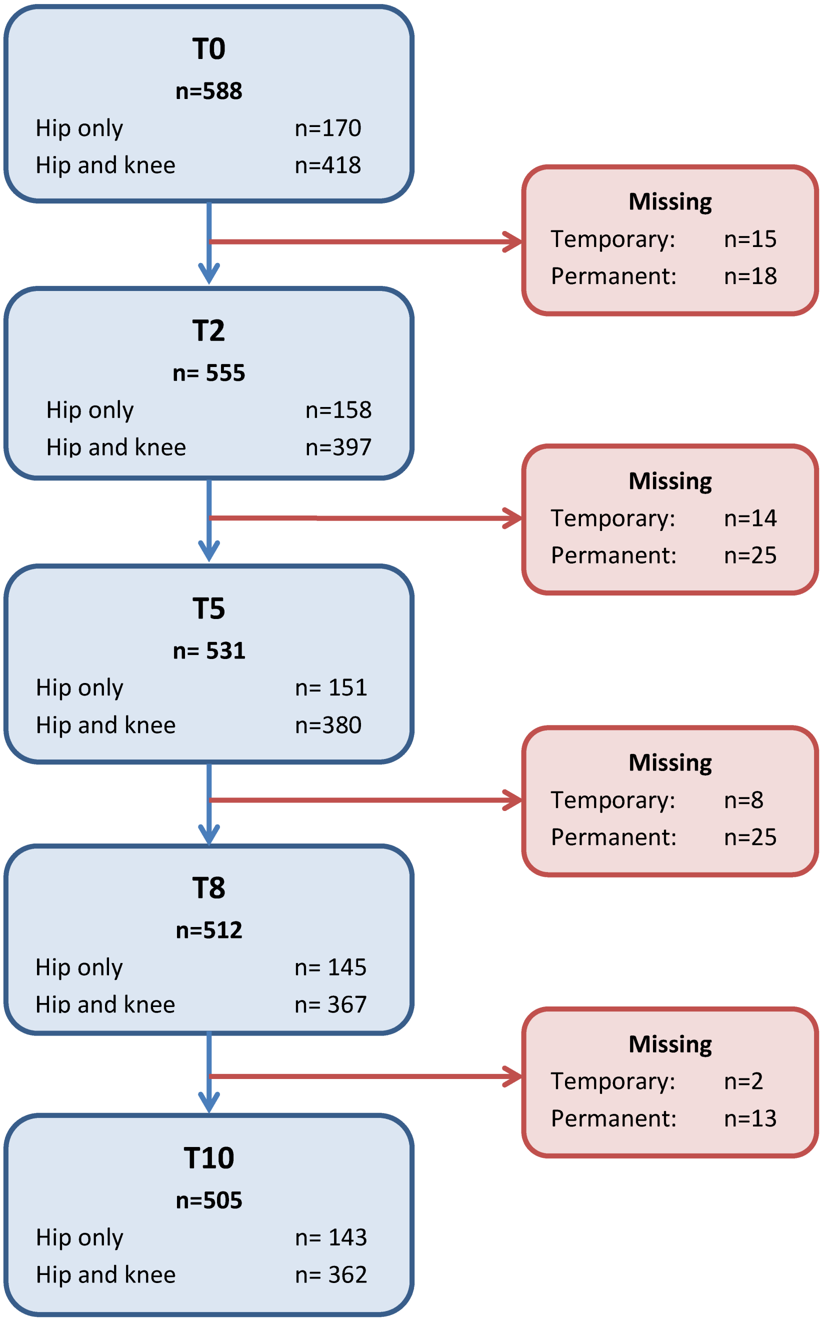

Supplementary Figure S1 Flow-chart indicating the available data per time point.

BMJ Publishing Group Limited (BMJ) disclaims all liability and responsibility arising from any reliance placed on this supplemental material which has been supplied by the author(s)

Supplementary Table S1. Course of pain, physical functioning, and radiographic OA features during follow-up, for total study group (n=588) and subdivided for H-group (n=170) and H&K-group (n=418).

	**Baseline**	**T2**	**T5**	**T8**	**T10**
WOMAC, mean (sd)					
**- Pain (0-20)**	5.4 (±3.4)	5.2 (±3.5)	5.1 (±4.0)	4.5 (±3.6)	4.7 (±3.8)
○ H-group	4.8 (±3.2)	4.3 (±3.5)	4.3 (±3.9)	3.5 (±3.4)	3.6 (±3.4)
○ H&K-group	5.7 (±3.5)	5.5 (±3.5)	5.4 (±4.0)	4.9 (±3.6)	5.1 (±3.9)
**- Stiffness (0-8)**	2.8 (±1.7)	2.6 (±1.6)	2.8 (±1.8)	2.4 (±1.8)	2.6 (±1.9)
○ H-group	2.5 (±1.7)	2.3 (±1.6)	2.5 (±1.8)	2.1 (±1.6)	2.1 (±1.8)
○ H&K-group	2.9 (±1.7)	2.7 (±1.6)	2.9 (±1.8)	2.5 (±1.8)	2.7 (±1.9)
**- Physical function (0-68)**	17.2 (±12.0)	16.4 (±12.0)	17.6 (±12.0)	16.3 (±13.0)	16.2 (±13.1)
○ H-group	14.7 (±11.1)	13.4 (±10.9)	14.3 (±12.8)	12.4 (±11.5)	12.2 (±10.7)
○ H&K-group	18.3 (±12.2)	17.6 (±12.3)	18.8 (±13.3)	17.8 (±13.2)	17.8 (±13.6)
**NRS past week, mean (sd)**	3.7 (±2.1)	3.7 (±2.3)	3.2 (±2.6)	2.7 (±2.5)	2.9 (±2.6)
○ H-group	3.4 (±2.2)	3.0 (±2.4)	3.0 (±2.6)	2.2 (±2.3)	2.5 (±2.5)
○ H&K-group	3.8 (±2.1)	4.0 (±2.2)	3.2 (±2.6)	2.9 (±2.5)	3.1 (±2.6)
**Use any pain medication, n (%)**	250 (43)	263 (48)	251 (47)	250 (49)	249 (50)
○ H-group	63 (38)	61 (39)	61 (40)	55 (38)	57 (40)
○ H&K-group	187 (46)	202 (52)	190 (50)	195 (53)	192 (54)
**Hip pain, n (%)**	588 (100)	374 (68)	301 (57)	267 (54)	247 (51)
○ H-group	170 (100)	114 (73)	90 (61)	71 (53)	57 (44)
○ H&K-group	418 (100)	260 (66)	211 (56)	196 (54)	190 (53)
**Knee pain, n (%)**	418 (71)	361 (65)	327 (62)	297 (58)	264 (53)
○ H-group	0 (0)	58 (37)	60 (40)	56 (39)	50 (37)
○ H&K-group	418 (100)	303 (77)	267 (70)	241 (66)	214 (60)
**Morning stiffness (hip) <60 min, n (%)**	326 (55)	287 (52)	272 (51)	239 (47)	228 (45)
○ H-group	101 (59)	85 (54)	77 (51)	61 (42)	58 (41)
○ H&K-group	225 (54)	202 (51)	195 (52)	178 (49)	170 (47)
**Physically active (>30 min) for ≥3 times a week, n (%)**	316 (55)	319 (60)	292 (56)	296 (58)	276 (56)
○ H-group	103 (62)	90 (58)	78 (52)	81 (57)	80 (56)
○ H&K-group	213 (53)	229 (60)	214 (57)	215 (59)	196 (56)
**Cumulative sum of HR, n (%)**	0 (0)	13 (2)	41 (7)	58 (10)	69 (12)
○ H-group	0 (0)	8 (5)	26 (15)	33 (19)	38 (22)
○ H&K-group	0 (0)	5 (1)	15 (4)	25 (6)	31 (7)
**Cumulative sum of KR, n (%)**	0 (0)	0 (0)	4 (1)	9 (2)	10 (2)
○ H-group	0 (0)	0 (0)	0 (0)	0 (0)	0 (0)
○ H&K-group	0 (0)	0 (0)	4 (1)	9 (2)	10 (2)
**K/L grade ≥2 either hip, n (%)**	110 (19)	128 (22)	151 (28)	183 (35)	253 (49)
○ **H-group**	38 (23)	40 (25)	48 (33)	54 (39)	64 (48)
○ **H&K-group**	72 (17)	88 (21)	103 (26)	129 (33)	189 (49)

**K/L grade ≥2 either knee, n (%)**	76 (13)	119 (20)	178 (37)	226 (51)	294 (69)
○ H-group	14 (8)	22 (14)	39 (26)	59 (42)	88 (63)
○ H&K-group	62 (15)	97 (25)	157 (42)	195 (55)	248 (70)
**Clinical OA* either knee, n (%)**	206 (35)	278 (47)	323 (55)	349 (59)	366 (62)
○ H-only	0 (0)	16 (9)	37 (22)	46 (27)	51 (30)
○ H&K	206 (50)	262 (63)	286 (68)	303 (73)	315 (75)
**Painful internal rotation either hip, n (%)**	322 (55)	197 (36)	190 (38)	166 (36)	179 (39)
○ H-only	101 (60)	61 (41)	51 (39)	43 (36)	49 (42)
○ H&K	221 (53)	136 (35)	139 (38)	123 (36)	129 (38)
**Painful external rotation either hip, n (%)**	160 (35)	86 (17)	115 (20)	86 (15)	89 (20)
○ H-only	45 (34)	28 (20)	31 (25)	26 (23)	22 (20)
○ H&K	115 (35)	58 (16)	84 (17)	60 (17)	67 (20)
**Painful flexion either hip, n (%)**	315 (54)	227 (42)	192 (39)	149 (32)	159 (35)
○ H-only	94 (56)	65 (43)	48 (38)	33 (29)	39 (35)
○ H&K	221 (54)	162 (41)	144 (39)	116 (33)	120 (35)

Values are: mean values ± the standard deviation, or number (percentages %). *according to the clinical criteria of the American College of Rheumatology; once those clinical ACR criteria are satisfied, the case will be seen as clinical hip or knee OA. NRS= Numeric Rating Scale (0-10). WOMAC = Western Ontario and McMaster osteoarthritis index. K/L= Kellgren and Lawrence score.

Supplementary Table S2. Course of pain, physical functioning, and radiographic OA features during follow-up, for the no HR-group (n=518) and HR-group (n=69) and for HR-group with LOCF.

	Baseline	T2	T5	T8	T10
WOMAC, mean (sd)					
**- Pain (0-20)**					
▪ No HR-group	5.4 (±3.4)	5.1 (±3.5)	5.3 (±4.0))	4.7 (±3.6)	4.9 (±3.8)
▪ HR-group	5.7 (±3.9)	5.5 (±3.7)	4.0 (±4.0)	3.6 (±3.8)	3.1 (±3.6)
▪ *✲HR-group with LOCF*	*5.7 (±3.9)*	*6.4 (±3.9)*	*6.9 (±4.1)*	*7.1 (±4.3)*	*7.1 (±4.3)*
**- Stiffness (0-8)**					
▪ No HR-group	2.8 (±1.7)	2.6 (±1.6)	2.9 (±1.8)	2.4 (±1.8)	2.6 (±1.9)
▪ HR-group	2.9 (±1.6)	2.6 (±1.7)	2.5 (±1.8)	2.1 (±1.8)	2.2 (±2.0)
▪ *✲HR-group with LOCF*	*2.9 (±1.6)*	*2.9* (±1.7)	*3.1* (±1.8)	*3.1 (±1.9)*	*3.1* (±1.9)
**- Physical function (0-68)**					
▪ No HR-group	16.9 (±11.9)	16.1 (±11.8)	17.8 (±13.3)	16.8 (±12.9)	16.9 (±13.1)
▪ HR-group	20.1 (±12.5)	18.5 (±13.6)	15.8 (±13.2)	13.3 (±13.5)	12.0 (±12.2)
▪ ***✲HR-group*** *with LOCF*	*20.1* (±12.5)	*21.1* (±13.7)	*23.9* (±13.9)	*24.9* (±14.6)	*24.9* (±14.6)
**NRS past week, mean (sd)**					
▪ No HR-group	3.6 (±2.1)	3.6 (±2.2)	3.2 (±2.6)	2.8 (±2.4)	3.0 (±2.7)
▪ HR-group	4.1 (±2.4)	4.1 (±2.6)	2.9 (±2.9)	2.3 (±2.5)	2.2 (±2.4)
▪ *✲HR-group with LOCF*	*4.1* (±2.4)	*4.6* (±2.5)	*4.9* (±2.7)	*5.1* (±2.7)	*5.1* (±2.7)
**Use any pain medication, n (%)**					
▪ No HR-group	221 (44)	227 (48)	222 (48)	220 (49)	224 (52)
▪ HR-group	29 (43)	35 (52)	28 (41)	29 (43)	24 (36)
▪ *✲HR-group with LOCF*	*29 (43)*	*36 (53)*	*43 (64)*	*45 (66)*	*45 (66)*
**Hip pain, n (%)**					
▪ No HR-group	518 (100)	326 (68)	266 (58)	239 (54)	231 (53)
▪ HR-group	69 (100)	47 (71)	35 (56)	27 (51)	15 (29)
▪ *✲HR-group with LOCF*	*69 (100)*	*59 (87)*	*59 (88)*	*61 (90)*	*61 (90)*
**Knee pain, n (%)**					
▪ No HR-group	386 (75)	328 (68)	295 (64)	265 (60)	240 (55)
▪ HR-group	31 (45)	32 (47)	32 (47)	31 (47)	23 (35)
▪ *✲HR-group with LOCF*	*31 (45)*	*34 (50)*	*32 (48)*	*34 (50)*	*34 (50)*
**Morning stiffness (hip) <60 min, n (%)**					
▪ No HR-group	282 (54)	244 (50)	236 (51)	210 (47)	202 (46)
▪ HR-group	43 (62)	42 (62)	35 (52)	28 (43)	25 (38)
▪ *✲HR-group with LOCF*	*43 (62)*	*48 (71)*	*49 (73)*	*51 (75)*	*51 (75)*
**Physically active (>30 min) for ≥3 times a week, n (%)**					
▪ No HR-group	282 (56)	283 (61)	257 (56)	256 (58)	239 (56)
▪ HR-group	34 (52)	35 (52)	35 (52)	40 (61)	37 (55)
▪ *✲HR-group with LOCF*	*34 (52)*	*34 (52)*	*35 (55)*	*38 (58)*	*38 (58)*
**Cumulative sum of HR, n (%)**					
▪ No HR-group	0 (0	0 (0)	0 (0)	0 (0)	0 (0)
▪ HR-group	0 (0)	13 (19)	41 (59)	58 (84)	69 (100)

**Cumulative sum of KR, n (%)**					
▪ No HR-group	0 (0)	0 (0)	4 (1)	8 (2)	9 (2)
▪ HR-group	0 (0)	0 (0)	0 (0)	1 (1)	1 (1)
**K/L grade ≥2 either hip, n (%)**					
▪ No HR-group	73 (14)	92 (20)	134 (30)	174 (39)	252 (59)
▪ HR-group	37 (54)	374 (66)	17 (61)	8 (73)	-
**K/L grade ≥2 either knee, n (%)**					
▪ No HR-group	64 (13)	103 (21)	166 (37)	221 (51)	293 (69)
▪ HR-group	12 (17)	16 (24)	30 (44)	33 (51)	42 (65)
**Clinical OA* either hip, n (%)**					
▪ No HR-group	125 (24)	171 (33)	205 (40)	226 (43)	243 (47)
▪ HR-group	34 (49)	43 (62)	51 (74)	54 (78)	54 (78)
**Clinical OA* either knee, n (%)**					
▪ No HR-group	194 (38)	253 (49)	296 (57)	320 (62)	335 (65)
▪ HR-group	12 (17)	24 (35)	26 (38)	28 (41)	30 (44)
**Painful internal rotation any hip, n (%)**					
▪ No HR-group	271 (53)	166 (34)	160 (35)	151 (34)	163 (37)
▪ HR-group	50 (73)	30 (55)	29 (74)	14 (70)	14 (78)
▪ *✡HR-group with LOCF*	*50 (73)*	*41 (60)*	*50 (75)*	*53 (79)*	*53 (79)*
**Painful external rotation any hip, n (%)**					
▪ No HR-group	131 (33)	69 (13)	101 (22)	77 (18)	80 (19)
▪ HR-group	28 (50)	17 (32)	13 (45)	8 (44)	8 (67)
▪ *✡HR-group with LOCF*	28 (50)	27 (42)	36 (56)	37 (58)	37 (58)
**Painful flexion any hip, n (%)**					
▪ No HR-group	265 (52)	199 (41)	174 (38)	138 (31)	146 (34)
▪ HR-group	48 (71)	28 (51)	17 (55)	17 (65)	12 (75)
▪ *✡HR-group with LOCF*	48 (71)	39 (57)	42 (63)	45 (67)	45 (67)

Values are: mean values ± the standard deviation, or number (percentages %). *according to the clinical criteria of the American College of Rheumatology; once those clinical ACR criteria are satisfied, the case will be seen as clinical hip or knee OA. NRS= Numeric Rating Scale (0-10). WOMAC = Western Ontario and McMaster osteoarthritis index. K/L= Kellgren and Lawrence score. ✡ *HR-group with LOCF:* for the participants of the HR-group we also show the observations with the last observation carried forward (LOCF) up to the last visit prior to HR.

Supplementary Table S3. Course of pain, physical functioning, and radiographic OA feature during follow-up with LOCF.

	Baseline	T2	T5	T8	T10
WOMAC, mean (sd)					
**- Pain (0-20)**	5.4 (±3.4)	5.3 (±3.6)	5.5 (±4.0)	5.0 (±3.8)	5.2 (±3.9)
○ H-group with LOCF for HR	4.8 (±3.2)	4.6 (±3.6)	5.1 (±3.9)	4.6 (±3.8)	4.8 (±3.7)
○ H&K-group with LOCF for HR	5.7 (±3.5)	5.6 (±3.6)	5.6 (±4.0)	5.1 (±3.7)	5.4 (±4.0)
**- Stiffness (0-8)**	2.8 (±1.7)	2.6 (±1.6)	2.9 (±1.8)	2.5 (±1.8)	2.7 (±1.9)
○ H-group with LOCF for HR	2.5 (±1.6)	2.4 (±1.6)	2.6 (±1.7)	2.4 (±1.8)	2.4 (±1.8)
○ H&K-group with LOCF for HR	2.9 (±1.7)	2.7 (±1.6)	3.0 (±1.8)	2.6 (±1.8)	2.8 (±1.9)
**- Physical function (0-68)**	17.2 (±12.0)	16.7 (±12.1)	18.6 (±13.5)	17.8 (±13.4)	18.0 (±13.6)
○ H-group with LOCF for HR	14.7 (±11.1)	14.2 (±11.4)	16.6 (±13.1)	15.8 (±12.9)	15.8 (±12.1)
○ H&K-group with LOCF for HR	18.3 (±12.2)	17.7 (±12.3)	19.3 (±13.6)	18.6 (±13.5)	18.8 (±14.0)
**NRS past week, mean (sd)**	3.7 (±2.1)	3.7 (±2.3)	3.4 (±2.7)	3.1 (±2.6)	3.3 (±2.8)
○ H-group with LOCF for HR	3.4 (±2.2)	3.2 (±2.3)	3.6 (±2.6)	3.1 (±2.7)	3.3 (±2.8)
○ H&K-group with LOCF for HR	3.8 (±2.1)	4.0 (±2.2)	3.4 (±2.7)	3.1 (±2.6)	3.4 (±2.7)
**Use any pain medication, n (%)**	250 (43)	264 (48)	266 (50)	266 (52)	270 (54)
○ H-group with LOCF for HR	63(38)	62 (40)	72 (48)	68 (47)	73 (51)
○ H&K-group with LOCF for HR	187 (46)	202 (52)	194 (51)	198 (54)	197 (55)
**Hip pain, n (%)**	588 (100)	386 (70)	325 (61)	301 (59)	293 (58)
○ H-group with LOCF for HR	170 (100)	121 (77)	108 (72)	96 (66)	85 (59)
○ H&K-group with LOCF for HR	418 (100)	265 (67)	217 (57)	205 (56)	208 (58)
**Knee pain, n (%)**	418 (71)	363 (66)	327 (62)	300 (59)	275 (55)
○ H-group with LOCF for HR	0 (0)	57 (36)	54 (36)	53 (36)	50 (35)
○ H&K-group with LOCF for HR	418 (100)	306 (77)	273 (72)	247 (68)	225 (63)
**Morning stiffness(hip)<60 min, n (%)**	326 (55)	293 (53)	286 (54)	262 (51)	254 (50)
○ H-group with LOCF for HR	101 (59)	90 (57)	87 (58)	78 (53)	75 (52)
○ H&K-group with LOCF for HR	225 (54)	203 (51)	199 (53)	184 (50)	179 (49)
**Physically active (>30 min) for ≥3 times a week, n (%)**	316 (55)	318 (60)	292 (56)	294 (58)	277 (56)
○ H-group with LOCF for HR	103 (62)	89 (58)	80 (54)	82 (57)	82 (58)
○ H&K-group with LOCF for HR	213 (53)	229 (60)	212 (57)	212 (58)	195 (55)
**Painful internal rotation either hip, n (%)**	322 (55)	208 (38)	211 (40)	205 (40)	217 (43)
○ H-only with LOCF for HR	101 (60)	68 (43)	67 (45)	67 (46)	73 (51)
○ H&K with LOCF for HR	221 (53)	140 (35)	477 (38)	138 (38)	144 (40)
**Painful external rotation either hip, n (%)**	160 (35)	96 (19)	138 (26)	115 (23)	118 (24)
○ H-group with LOCF for HR	45 (34)	34 (23)	45 (30)	44 (31)	39 (28)
○ H&K-group with LOCF for HR	115 (28)	62 (17)	93 (25)	71 (20)	79 (22)
**Painful flexion either hip, n (%)**	315 (54)	238 (43)	217 (41)	183 (36)	192 (38)
○ H H-group with LOCF for HR	94 (56)	72 (46)	65 (38)	57 (39)	61 (42)
○ H&K-group with LOCF for HR	221 (54)	166 (42)	152 (40)	126 (34)	131 (36)

Values are: mean values ± the standard deviation, or number (percentages %). *according to the clinical criteria of the American College of Rheumatology; once those clinical ACR criteria

are satisfied, the case will be seen as clinical hip or knee OA. NRS= Numeric Rating Scale (0-10). WOMAC = Western Ontario and McMaster osteoarthritis index. K/L= Kellgren and Lawrence score. *With LOCF for HR:* for the participants who received an HR we show the observations with the last observation carried forward (LOCF) up to the last visit prior to HR.
